# Preoperative differentiation of acute appendicitis using NLR, SII, and maximum appendiceal diameter: a retrospective single-center diagnostic study

**DOI:** 10.3389/fsurg.2026.1795204

**Published:** 2026-04-01

**Authors:** Xu Junyan, Zhang Tao

**Affiliations:** 1 Chengde Medical University, Chengde, Hebei, China; 2 Department of General Surgery, The No.2, Hospital of Baoding, Baoding, Hebei, China

**Keywords:** acute appendicitis, maximum appendiceal diameter, neutrophil-to-lymphocyte ratio, preoperative classificatio, systemic immune-inflammation index

## Abstract

**Background:**

Rapid classification on Accurate preoperative differentiation between uncomplicated (UCAA) and complicated acute appendicitis (CAA) is essential for guiding treatment but remains challenging. Existing tools, such as clinical scores and common inflammatory markers, lack sufficient accuracy for reliable stratification.

**Methods:**

In this retrospective single-center study, 207 patients with pathologically confirmed appendicitis (52 UCAA, 155 CAA) undergoing laparoscopic appendectomy between June 2024 and September 2025 were analyzed. Multivariate logistic regression and ROC curve analyses were performed.

**Results:**

Individual AUCs were 0.863 for NLR (cut-off 5.22), 0.803 for SII (cut-off 1292.3), and 0.815 for maximum appendiceal diameter (cut-off 10.5 mm). Their combination achieved a superior AUC of 0.891 (95% CI: 0.840–0.942), with 86.54% sensitivity and 80.65% specificity. NLR and maximum appendiceal diameter were independent predictors of CAA.

**Conclusion:**

The combination of NLR, SII, and maximum appendiceal diameter improves preoperative diagnostic accuracy for acute appendicitis over single parameters, potentially aiding individualized treatment planning and reducing diagnostic uncertainty.

## Introduction

Acute appendicitis (AA) is a common acute abdominal condition in emergency surgery (1). According to clinical guidelines, AA is classified into two main categories based on clinical symptoms or histopathological findings, uncomplicated acute appendicitis (UCAA) and complicated acute appendicitis (CAA). The World Society of Emergency Surgery (WSES) defines CAA as cases that have progressed to perforation, gangrene, or abscess formation. In contrast, UCAA is characterized by acute inflammation and congestion of the appendiceal wall, without necrosis or abscess (2). This classification serves as the fundamental framework for case categorization in this statistical study.

Epidemiological surveys indicate that in developed countries, AA predominantly affects individuals aged 10–30 years (3, 4). The lifetime risk is higher in males (8.6%) than in females (6.7%), yet the lifetime appendectomy rate is significantly higher in females (23%) compared to males (12%) (5). Globally, the annual incidence ranges from approximately 96.5–100 cases per 100,000 adults. While AA mortality has been declining, its incidence shows a concerning upward trend (6).

Since the 18th century, appendectomy has been the standard treatment (7–9). By the 1980s, laparoscopic appendectomy, with advantages such as minimal invasiveness, faster recovery, shorter hospital stays, and lower infection rates, became the preferred surgical approach for AA, and its use has increased substantially (10). However, the high volume of procedures has also led to a significant negative appendectomy rate (11). Early surgery, performed before inflammation is fully developed, may contribute to this rate. Conversely, delayed surgery increases the risk of perforation and other complications. With evolving research, treatment is no longer limited to surgery. Studies show that antibiotic therapy alone can successfully treat UCAA. One study comparing open appendectomy with antibiotic therapy reported complication rates of 20.5% and 24.4% in the surgical group at 1 and 5 years, respectively, vs. only 2.8% and 6.5% in the antibiotic group (12). A prospective multicenter randomized controlled trial further supports antibiotics as an acceptable treatment for patients with uncomplicated appendicitis (13). In contrast, CAA requires prompt appendectomy to prevent disease progression and serious complications (3). Therefore, determining the appropriate treatment strategy and surgical timing is critical. However, a predictive model that can early, accurately, and conveniently distinguish CAA from UCAA is still lacking.

AA diagnosis relies on a combination of history, physical examination, laboratory tests, and imaging. Scoring systems like the Alvarado score and the Appendicitis Inflammatory Response (AIR) score can aid diagnosis (3, 14, 15). However, no single indicator currently offers both high sensitivity and specificity for diagnosing AA and simultaneously classifying its type. Commonly used inflammatory markers like white blood cell (WBC) count and C-reactive protein (CRP) play key roles in phagocytic activity and inflammation regulation but lack sufficient predictive value for AA classification on their own (16, 17).

Beyond these, the Neutrophil-to-Lymphocyte Ratio (NLR) and the Systemic Immune-Inflammation Index (SII) have emerged in recent research as cost-effective, readily accessible, and valuable inflammatory markers (18, 19). Neutrophilia coupled with lymphocytopenia is a hallmark of systemic inflammatory response; a greater disparity indicates more severe inflammation. Consequently, NLR is widely used to assess inflammation in various diseases (20). SII has also been studied for evaluating conditions like cardiovascular diseases, metabolic disorders, and ICU patient prognosis (20, 21). Both NLR and SII reflect changes in immune and inflammatory pathways. Research on these indices has grown significantly over the past decade, accumulating substantial evidence for their potential as tools to predict AA severity in adults, though their utility for rapid AA classification remains unexplored. Imaging, particularly computed tomography (CT), is commonly used to confirm AA (22). CT is fast, widely available even in primary care hospitals, and offers greater accuracy, lower cost, and faster diagnosis compared to MRI or ultrasound for AA. However, CT alone has limited sensitivity and may miss some CAA cases, potentially delaying surgery. This study aims to combine imaging and hematological parameters to evaluate their potential value in distinguishing UCAA from CAA.

In summary, the clinical presentation of AA spans a broad spectrum, from simple appendiceal wall inflammation to perforation with localized or diffuse peritonitis. Therefore, this study investigates the potential utility of the NLR, SII, and/or maximum appendiceal diameter in differentiating UCAA from CAA. We aim to assess whether these three variables can enable a quicker and more reliable differentiation to guide treatment decisions, hoping this approach can provide new indicators for clinical management strategies.

## Method

### Research design

This was a retrospective, cross-sectional, single-center study. Adult patients diagnosed with acute appendicitis who underwent laparoscopic appendectomy between June 2024 and September 2025 were enrolled. Based on postoperative pathology findings, AA cases were categorized into two groups: CAA and UCAA.

### Data collection

The collected data included: (1) demographic characteristics, such as gender and age; (2) preoperative routine blood test parameters, including white blood cell count, lymphocyte count, platelet count, neutrophil count, monocyte count, red blood cell distribution width (RDW), the calculated SII, and the NLR; and (3) postoperative pathological classification.

### Study population

**Figure 1:**
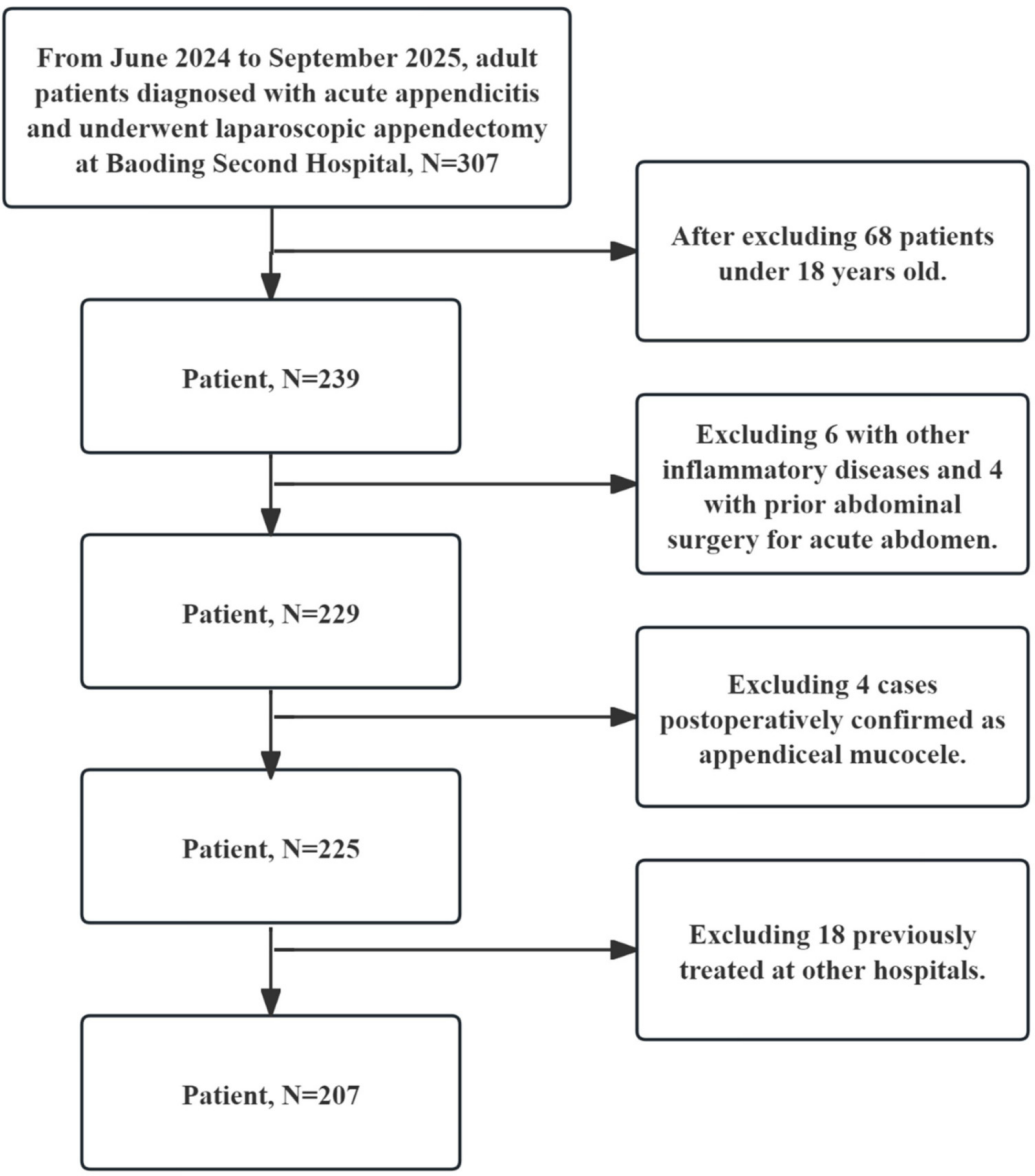
Flowchart of participant inclusion and exclusion by research staff.

A total of 307 patients with histologically confirmed AA were initially assessed. Subsequently, 68 patients under 18 years of age, 6 patients with concomitant inflammatory diseases, 4 patients with a history of previous abdominal surgery for acute abdomen, and 4 patients diagnosed with appendiceal mucocele based on postoperative pathology were excluded. Additionally, 18 patients who had received prior treatment at other institutions were also excluded. Consequently, 207 patients were ultimately included in this study. The schematic diagram is shown in Figure 1.

### Primary and secondary outcomes

This study aimed to determine whether the NLR, SII, and maximum appendiceal diameter can differentiate between UCAA and CAA, and to evaluate whether their combined use improves the reliability of diagnosing UCAA vs. CAA. A secondary objective was to promote individualized treatment by enabling early preoperative classification, thereby reducing the negative appendectomy rate and avoiding unnecessary surgical interventions.

### Ethics statement

This study was conducted in accordance with the principles of the Declaration of Helsinki and was approved by the Institutional Review Board of our hospital (No. CY2026001). The requirement for informed consent was waived due to the retrospective nature of the study.

### Statistical analysis

Data analysis was performed using SPSS Statistics 26.0 (IBM Corp., Armonk, NY, USA). Receiver operating characteristic (ROC) curves were plotted using the ggplot2 package in R language, while nomograms were constructed and fitted using the rms and ResourceSelection packages in R. Patient characteristics were summarized using descriptive statistics: normally distributed variables are presented as mean ± standard deviation, and non-normally distributed variables as median with interquartile range. Categorical data are described as frequencies and percentages (%). Comparisons between the UCAA and CAA groups were conducted as follows: independent samples t-test for normally distributed continuous variables, Mann–Whitney *U* test for non-normally distributed continuous variables, and Chi-square test or Fisher's exact test for categorical variables, as appropriate. Variables showing statistically significant differences in univariate analyses were included in a multivariate logistic regression model to assess the independent associations of SII, NLR, and maximum appendiceal diameter with CAA, and to develop a predictive model. Receiver operating characteristic (ROC) curves were plotted to evaluate the diagnostic accuracy of these indicators—both individually and in combination—in distinguishing CAA from UCAA. The area under the curve (AUC) was interpreted as follows: 0.9–1.0, excellent; 0.8–0.9, good; 0.7–0.8, acceptable.

## Result

During the period from June 2024 to September 2025, a total of 307 patients diagnosed with acute appendicitis who underwent laparoscopic appendectomy were initially identified and evaluated. The following exclusions were applied: 68 patients under 18 years of age, 5 patients with renal failure, 3 patients with tumors, 4 patients with pathologically confirmed appendiceal mucocele, 2 patients who underwent open appendectomy, and 18 patients who had received prior treatment at other institutions. Consequently, 207 patients were ultimately included in this study. Among the included cases, there were 102 males (49.2%) and 105 females (50.7%). The mean age was 42.006 ± 15.083 years in the complicated appendicitis group and 37.981 ± 14.098 years in the uncomplicated appendicitis group.

Based on postoperative pathology, patients were classified into UCAA and CAA groups. According to the data, 155 patients (74.9%) were diagnosed with CAA, while the remaining 52 patients (25.1%) had UCAA. Compared to the UCAA group, patients in the CAA group exhibited significantly higher median values of SII, NLR, SIRI, MLR, and RDWLR, alongside significantly lower median platelet count, absolute lymphocyte count, and eosinophil count. Furthermore, statistically significant differences between the two groups were also observed in terms of white blood cell count, absolute neutrophil count, and maximum appendiceal diameter (Table 1).

**Table 1:** Comparison of baseline data between the two groups.

Characteristics	CAA	UCAA	Method
*N*	155	52	
Gender, *n* (%)			Chisq test
Male	75 (36.2%)	27 (13.1%)	
Female	80 (38.6%)	25 (12.1%)	
Age, mean ± sd	42.006 ± 15.083	37.981 ± 14.098	*T* test
SII, median (IQR)	1,871.3 (1,193.5, 2,424.2)	923.54 (621.86, 1,256.9)	Wilcoxon
SIRI, median (IQR)	4.3699 (2.8065, 6.8419)	2.1284 (1.1361, 2.9637)	Wilcoxon
RDWLR, median (IQR)	10.84 (7.6878, 13.886)	6.897 (5.4759, 8.6777)	Wilcoxon
RDW, median (IQR)	12.6 (12.2, 13.1)	12.7 (12.4, 13)	Wilcoxon
Mon, median (IQR)	0.54 (0.37, 0.67)	0.49 (0.395, 0.645)	Wilcoxon
MLR, median (IQR)	0.40351 (0.31232, 0.55271)	0.27848 (0.20496, 0.3612)	Wilcoxon
PLR, median (IQR)	163.33 (123.5, 220.67)	138.09 (103.88, 162.15)	Wilcoxon
PLT, median (IQR)	201 (161.5, 234.5)	245 (207.5, 279)	Wilcoxon
EOS, median (IQR)	0.02 (0, 0.05)	0.06 (0.0375, 0.11)	Wilcoxon
ELR, median (IQR)	0.014778 (0, 0.040931)	0.032976 (0.020462, 0.062172)	Wilcoxon
WBC, mean ± sd	12.653 ± 3.5711	9.7956 ± 2.9373	T test
NEU, mean ± sd	10.819 ± 3.355	7.2473 ± 2.5873	T test
LYM, median (IQR)	1.2 (0.94, 1.655)	1.85 (1.4425, 2.325)	Wilcoxon
NLR, median (IQR)	9.1869 (6.4951, 12.071)	3.9744 (2.9309, 4.9493)	Wilcoxon
Maximal appendiceal diameter, median (IQR)	12 (10, 13)	8 (7, 10)	Wilcoxon

Univariate and multivariate logistic regression models were constructed, incorporating variables such as sex, age, and SII. A total of 16 hematological and imaging indicators were evaluated. Univariate analysis revealed that, apart from sex, red cell distribution width (RDW), monocyte count (Mon), and eosinophil-to-lymphocyte ratio (ELR), the remaining 13 variables—including SII, SIRI, NLR, PLR, platelet count (PLT), white blood cell count (WBC), neutrophil count (NEU), lymphocyte count (LYM), eosinophil count (EOS), RDWLR, age, and maximum appendiceal diameter—were significantly associated with UCAA (*P* < 0.1), thereby meeting the criteria for inclusion in the multivariate logistic regression analysis. In the multivariate logistic regression model, a smaller maximum appendiceal diameter and a lower NLR value were identified as independent factors associated with a higher likelihood of UCAA (Table 2).

**Table 2:** Univariate and multivariate regression analysis results.

Characteristics	Total (*N*)	OR(95% CI) univariate analysis	*P* value univariate analysis	OR(95% CI) multivariate analysis	*P* value multivariate analysis
Gender	207				
Male	102				
Female	105	0.868 (0.463–1.628)	0.659		
Age	207	0.981 (0.960–1.003)	0.094	0.991 (0.959–1.025)	0.608
SII	207	0.998 (0.998–0.999)	<0.001	0.996 (0.992–1.001)	0.104
SIRI	207	0.566 (0.454–0.706)	<0.001	0.269 (0.065–1.109)	0.069
RDWLR	207	0.834 (0.758–0.918)	<0.001	0.925 (0.755–1.133)	0.451
RDW	207	0.970 (0.850–1.108)	0.657		
Mon	207	0.623 (0.165–2.352)	0.485		
PLR	207	0.992 (0.987–0.998)	0.006	0.993 (0.967–1.019)	0.603
PLT	207	1.012 (1.006–1.018)	<0.001	1.035 (1.011–1.058)	0.003
EOS	207	61.241 (3.483–1,076.806)	0.005	0.384 (0.003–51.901)	0.702
ELR	207	9.655 (0.306–304.275)	0.198		
WBC	207	0.773 (0.694–0.862)	<0.001	2.080 (0.725–5.966)	0.173
NEU	207	0.683 (0.599–0.779)	<0.001	0.467 (0.134–1.627)	0.232
LYM	207	3.997 (2.294–6.966)	<0.001	0.560 (0.083–3.756)	0.55
NLR	207	0.644 (0.556–0.745)	<0.001	2.086 (1.147–3.791)	0.016
Maximal appendiceal diameter	207	0.564 (0.467–0.681)	<0.001	0.660 (0.523–0.831)	<0.001

**Figure 2:**
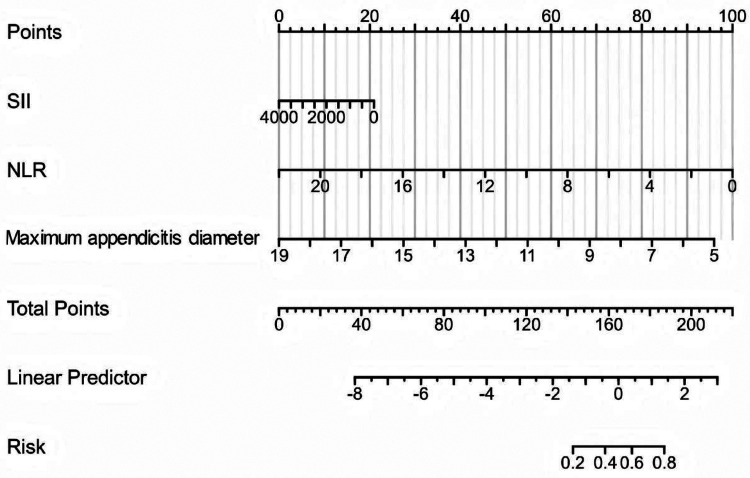
Acute appendicitis subtype prediction diagram.

Based on screening of the three optimal predictive variables, a nomogram for acute appendicitis subtype prediction was constructed, as shown in Figure 2. The figure displays the contribution of each variable. Specific points are assigned corresponding to the value of each option, and the total points are obtained by summing the points from all three variables. The bottom of Figure 2 provides the probability prediction corresponding to different total point scores. A higher total score indicates a greater likelihood of uncomplicated acute appendicitis.

For example, consider a patient whose SII is 1,000, NLR is 4, and maximal appendiceal diameter is 6 mm. Using the nomogram, the corresponding points are calculated as 15, 83, and 89 points, respectively, resulting in a total score of 187 points and a predicted probability of 0.7 for uncomplicated appendicitis. Therefore, this patient can be considered to have a high likelihood of uncomplicated acute appendicitis with low risk of adverse outcomes, and conservative management or close observation may be considered.

**Figure 3:**
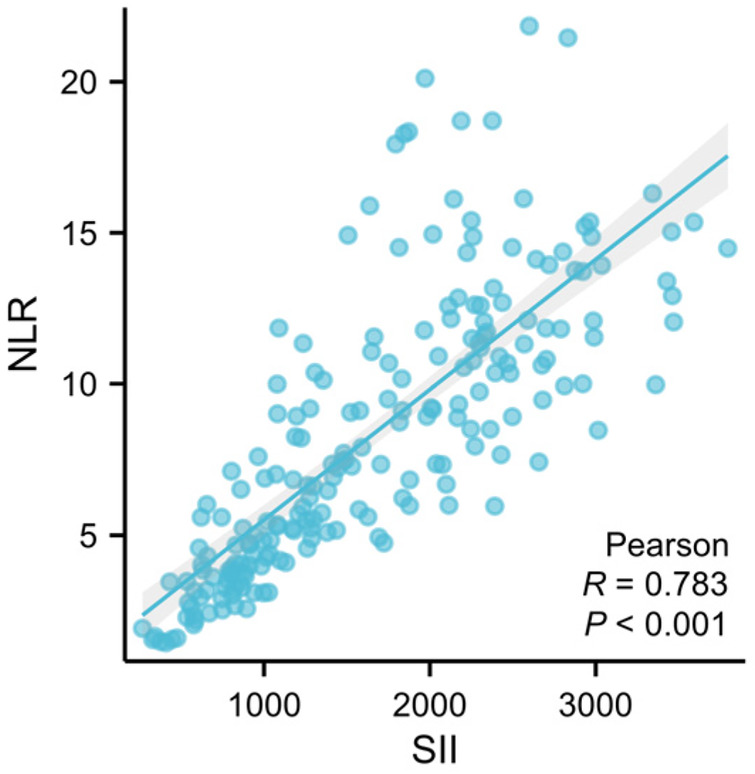
Moderate positive correlation between NLR and SII (*R* = 0.783, *P* < 0.001).

Given that both NLR and SII are calculated from neutrophil and lymphocyte counts, we performed Pearson correlation analysis to assess the degree of collinearity between these two indices. Scatter plots were generated and correlation coefficients were calculated using SPSS 26.0 (IBM Corp., Armonk, NY, USA), with the significance level set at *α* = 0.05. The Pearson correlation analysis (Figure 3) revealed a moderate positive correlation between NLR and SII (*r* = 0.783, *P* < 0.001). Multicollinearity diagnostics showed VIF values (Table 3) of 2.581 for NLR and 2.581 for SII, which are well below the conventional threshold of 5. This indicates that although the two indices share common components, the degree of collinearity is acceptable and does not compromise the stability of the regression coefficients.

**Table 3:** Multicollinearity Test.

Variable	VIF	1/VIF
SII	2.581	0.387
NLR	2.581	0.387
Mean VIF	2.581	

**Figure 4:**
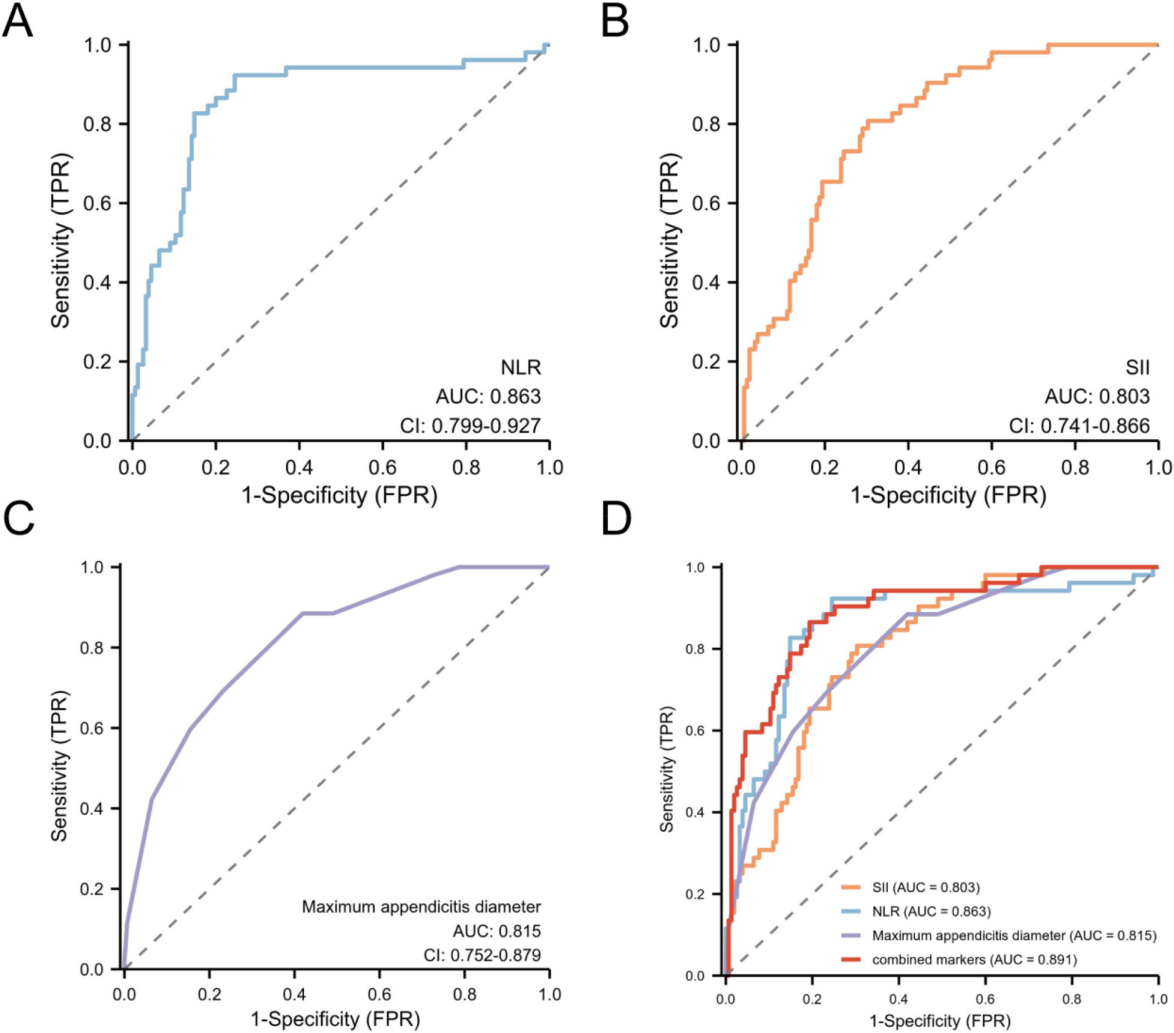
ROC curve of the study sample.

ROC analysis was performed using SPSS 26.0 (IBM Corp., Armonk, NY, USA). Receiver operating characteristic (ROC) curves were plotted with 1-specificity on the *x*-axis and sensitivity on the *y*-axis (Figure 4). The area under the curve (AUC) was used to evaluate the reliability of each indicator in differentiating between uncomplicated and complicated acute appendicitis.

The results showed that the NLR yielded an AUC of 0.863 (95% CI: 0.799–0.927). The systemic SII and maximum appendiceal diameter also demonstrated good diagnostic performance, with AUCs of 0.803 (95% CI: 0.741–0.866) and 0.815 (95% CI: 0.752–0.879), respectively. The combination of all three indicators achieved the highest AUC of 0.891 (95% CI: 0.840–0.942).

**Figure 5:**
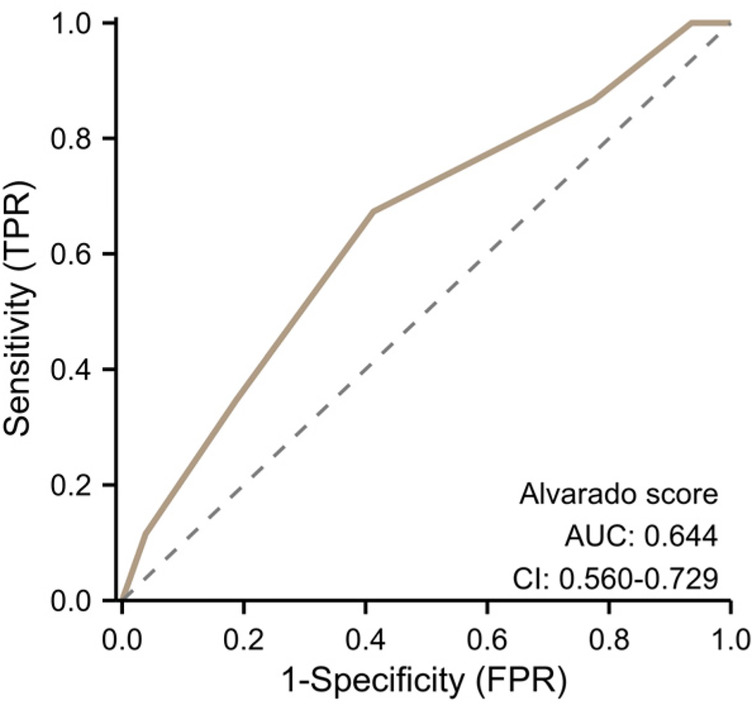
ROC curve of the Alvarado score.

As shown in Table 4, the optimal cut-off value for SII was 1,292.3, yielding a sensitivity of 80.77% and a specificity of 69.69%. Specificity represents the proportion of true negatives among all actual uncomplicated cases; thus, 69.69% of patients with pathologically confirmed uncomplicated appendicitis have SII values below 1,292.3. For NLR, the cut-off value was 5.22, with a sensitivity of 82.69% and a specificity of 85.16%. Sensitivity represents the proportion of true positives among all actual complicated cases; thus, 82.69% of patients with complicated appendicitis are correctly identified by NLR >5.22. Regarding the maximum appendiceal diameter, the cut-off was 10.5 mm, achieving a sensitivity of 88.46% and a specificity of 58.07%. This indicates that 88.46% of complicated appendicitis cases have diameters >10.5 mm. However, when the three markers were combined for disease prediction, the integration of SII, NLR, and maximum appendiceal diameter demonstrated a significant linear relationship and enhanced diagnostic performance.To compare with classical clinical scoring systems, we performed ROC analysis of the Alvarado score in all 207 patients (Figure 5). The results showed that the Alvarado score achieved an AUC of 0.644 (95% CI: 0.560–0.729) for differentiating CAA from UCAA, with a sensitivity of 58.7% and specificity of 62.5%, which was markedly inferior to our combined model (AUC: 0.891, sensitivity 86.54%, specificity 80.65%). These findings indicate that integrating objective laboratory and imaging parameters (NLR, SII, and maximal appendiceal diameter) provides significantly superior diagnostic performance compared to the traditional Alvarado score, which relies on clinical symptoms and physical examination findings, thereby offering more reliable decision support for rapid and accurate preoperative classification of acute appendicitis.

**Table 4:** Relevant indicators of the study sample.

Index	Best cut-off	Sensitivity	Specificity	AUC (95%*CI*)
SII	1,292.30	0.8077	0.6968	0.803
NLR	5.22	0.8269	0.8516	0.863
Maximal appendiceal diameter	10.50	0.8846	0.5807	0.815
Combined diagnosis	−1.10	0.8654	0.8065	0.891

## Discussion

Accurate preoperative classification of acute appendicitis remains a central challenge in modern emergency surgery for achieving individualized treatment (23–25). Although laparoscopic appendectomy has become the gold standard, clinicians continue to face the difficult dilemma of balancing the risk of negative appendectomy due to overtreatment against the risk of perforation from delayed surgery. Existing scoring systems, such as the Alvarado and Appendicitis Inflammatory Response (AIR) scores, are simple and practical but have limited efficacy in differentiating between UCAA and CAA. Currently, there is a lack of a rapid, accurate, and easily applicable preoperative prediction model. To address this, this retrospective study systematically evaluated the diagnostic value of combining the NLR, the SII, and maximum appendiceal diameter in distinguishing UCAA from CAA.

The results clearly demonstrate that NLR, SII, and maximum appendiceal diameter each possess a certain discriminatory capacity, with AUC values of 0.863, 0.803, and 0.815, respectively. When combined, their diagnostic performance improved significantly, achieving a combined AUC of 0.891, which was superior to any single indicator, highlighting the advantage of the integrated model in comprehensive discrimination. Multivariate logistic regression analysis further confirmed that NLR and maximum appendiceal diameter are independent predictors of CAA. Although SII did not emerge as an independent predictor in this study, it still contributed diagnostic information to the combined model. Its value is consistent with previous literature; for instance, a systematic review by Arredondo et al. indicated that SII can achieve an AUC of up to 0.85 in diagnostic classification, suggesting its strong clinical potential as an inflammatory marker derived from routine complete blood count parameters (26).

From the perspective of pathological mechanisms, the effectiveness of this combined model stems from its comprehensive reflection of the core inflammatory processes in CAA (27, 28). CAA is not only characterized by local tissue destruction but is also accompanied by a systemic immune-inflammatory imbalance. The NLR captures both the pro-inflammatory state driven by neutrophils and the immunoregulatory function represented by lymphocytes. Its significantly elevated level in the CAA group directly reflects this imbalance. The strength of the SII lies in its further integration of platelet count, as platelet activation is a key driver of tissue necrosis. In severe inflammation, platelets not only amplify the inflammatory response but may also contribute to local tissue ischemia through microthrombus formation, thereby significantly increasing the risk of gangrene or perforation. In this study, the median values of both NLR and SII were significantly higher in the CAA group than in the UCAA group, which aligns with the conclusions of several recent meta-analyses and further validates their biological plausibility in inflammation assessment (26, 29, 30).

Regarding imaging, while maximum appendiceal diameter can visually indicate local edema and exudation, its specificity is low when used alone and is susceptible to individual variation and measurement error (31). By integrating systemic inflammatory markers with local morphological changes, this study improved diagnostic specificity from 58.07% (relying solely on diameter) to 80.65%, effectively overcoming the limitations of single-method approaches and enhancing the overall accuracy of preoperative assessment (22).

In terms of clinical translation, the nomogram constructed based on the above variables offers good visualization and individualized predictive capability, making it particularly suitable for aiding decision-making in time-sensitive settings such as the emergency department. For example, patients with a predicted probability ≤0.25 (low risk) may be considered for conservative management, which is expected to significantly reduce the negative appendectomy rate and associated surgical risks (12). Conversely, for patients with a probability ≥0.75 (high risk), active surgical intervention is recommended to minimize the risk of perforation and other complications (32). This strategy provides a practical tool for achieving individualized and precise management of acute appendicitis, with the potential to optimize clinical resource allocation while ensuring patient safety.

Compared with models such as the AAS grade or RIPASA score, which rely on subjective clinical signs, the strength of this study lies in its complete dependence on objective laboratory and imaging parameters, thereby avoiding inter-observer variability in physical examination and patient-reported bias (33–35). In contrast to emerging radiomics or machine-learning models, the acquisition of NLR, SII, and maximum appendiceal diameter requires no complex algorithms or high costs, making it readily applicable even in primary healthcare settings and thus offering broader generalizability. It should be emphasized that this model is not intended to completely replace clinical judgment, but rather to serve as a standardized auxiliary tool that provides emergency physicians with quantitative decision-making support and reduces the arbitrariness of empirical treatment.

It must be acknowledged that, as a single-center retrospective analysis, this study inevitably carries limitations such as selection bias. Although internal validation demonstrated satisfactory model performance, its generalizability requires further external validation through prospective, multicenter studies. Furthermore, future research could integrate additional clinical variables such as symptom duration and comorbidities, or explore novel biomarkers, to continually improve the predictive accuracy and clinical applicability of the model.

## Conclusion

This study systematically investigated the diagnostic value of the NLR, SII, and maximum appendiceal diameter in preoperatively differentiating between uncomplicated and complicated acute appendicitis, providing a practical classification prediction tool for clinicians in primary care settings. The individualized risk assessment offered by this prediction model enables more accurate diagnostic classification, which may inform subsequent treatment planning, thereby effectively reducing the negative appendectomy rate and the risk of complications, and ultimately improving overall patient prognosis. It should be noted that this study is a single-center retrospective analysis with a relatively limited sample size. As we only included patients who underwent laparoscopic appendectomy, excluding cases managed conservatively with antibiotics or transferred to other institutions, there is inherent selection bias. This selection limits the direct applicability of our model to the critical clinical decision point of operative vs. non-operative management. Although our predictive model can accurately differentiate between uncomplicated and complicated appendicitis, the decision to pursue conservative treatment still requires integration of other clinical factors. Furthermore, we did not incorporate clinical variables such as symptom duration due to incomplete documentation in our center's electronic medical record system, which relied heavily on patient recall and was often unreliable or poorly documented in emergency department records. Future prospective studies should prioritize standardized collection of symptom duration and other clinical time parameters to enhance predictive accuracy. The proportion of complicated appendicitis in this study was markedly higher than that reported in the general population, suggesting potential spectrum bias. To eliminate this spectrum bias, we will conduct multicenter prospective cohort validation across different levels of medical institutions. Future research should focus on conducting multicenter, prospective cohort validations, exploring the integration of additional inflammatory markers or imaging features, and assessing the model's utility in guiding treatment decisions including conservative management to continuously enhance the accuracy and generalizability of the prediction model.

## Data Availability

The raw data supporting the conclusions of this article will be made available by the authors, without undue reservation.
